# A case report of Grover’s disease from immunotherapy-a skin toxicity induced by inhibition of CTLA-4 but not PD-1

**DOI:** 10.1186/s40425-016-0157-6

**Published:** 2016-09-20

**Authors:** Marc Uemura, Faa’k Faisal, Cara Haymaker, Natalie McQuail, Elizabeth Sirmans, Courtney W. Hudgens, Lydia Barbara, Chantale Bernatchez, Jonathan L. Curry, Patrick Hwu, Michael T. Tetzlaff, Adi Diab

**Affiliations:** 1 Department of Melanoma Medical Oncology, University of Texas-MD Anderson Cancer Center, Houston, TX USA; 2 Department of Pathology, University of Texas-MD Anderson Cancer Center, Houston, TX USA

## Abstract

**Background:**

Immune related adverse events (irAEs) are common side effects of checkpoint inhibitory (CPI) therapies targeting CTLA-4 and PD-1/PD-L1. Grover’s disease is an uncommon dermatologic condition with unclear pathogenesis previously reported as an irAE with ipilimumab.

**Case Presentation:**

We report an additional case of ipilimumab-induced Grover’s disease. Interestingly, this dermatologic side effect did not appear with use of anti-PD-1 therapy in our patient. Immune analysis was performed and suggests a possible role of Th2 cells in its patholgenesis.

**Conclusion:**

This case suggests that Grover's disease is an irAE induced by Ipilimumab. Our immune analysis suggests that Th2 cells may be pathogenic mediators which warrants further study.

## Background

Grover’s Disease, or transient acantholytic dermatosis, was first described in 1970 in 6 patients presenting with an unusual pruritic, papulovesicular dermatosis [1]. Thereafter, retrospective studies described the disease’s distinct clinicopathologic features. Predominantly on the trunk, back and extremities in adults over 40 years of age, the distinctive skin lesions typically present with a variety of histologic patterns (like pemphigus, Darier, and Hailey-Hailey) that mimic other acantholytic dermatoses [2, 3]. Given its rarity, the disease’s precise etiology and pathogenesis remains unclear but studies have suggested numerous entities, including drugs (recombinant IL-4; RAF inhibitors), solid and hematologic malignancies, organ transplantation, ultraviolent exposure and heat [4–7]. Recently, Grover’s disease was reported in a patient with metastatic melanoma treated with ipilimumab [8].

Here, we report another case of ipilimumab-induced Grover’s disease and provide a possible immunological mechanism underlying the disease’s pathogenesis in relationship to CTLA-4, but not PD-1 inhibition. To our knowledge, there are no other cases describing this immunologic mechanism for ipilimumab-induced Grover’s disease in the medical literature.

## Case presentation

A 73 y/o male presented with stage IIA cutaneous melanoma of the left shoulder and underwent surgical resection in 2003. In November 2012, he developed bilateral lung nodules with no other metastatic sites. A right lung biopsy at this time confirmed metastatic melanoma. He initiated therapy with ipilimumab at 3 mg/kg IV every three weeks. After his second treatment dose, he developed an intensely pruritic, papulovesicular rash over his chest, bilateral upper extremities, and back. Prior to this, the patient had no history of skin rashes. He received methylprednisolone (40 mg IV initially, then 20 mg IV 1 month later without topical therapy) with significant pruritus improvement; however, the rash persisted. His outside physician then discontinued ipilimumab in March 2013 because of persistent rash symptoms and transitioned the patient to close observation. Remarkably, his lung nodules remained stable based on CT imaging performed every six months.

**Fig. 1:**
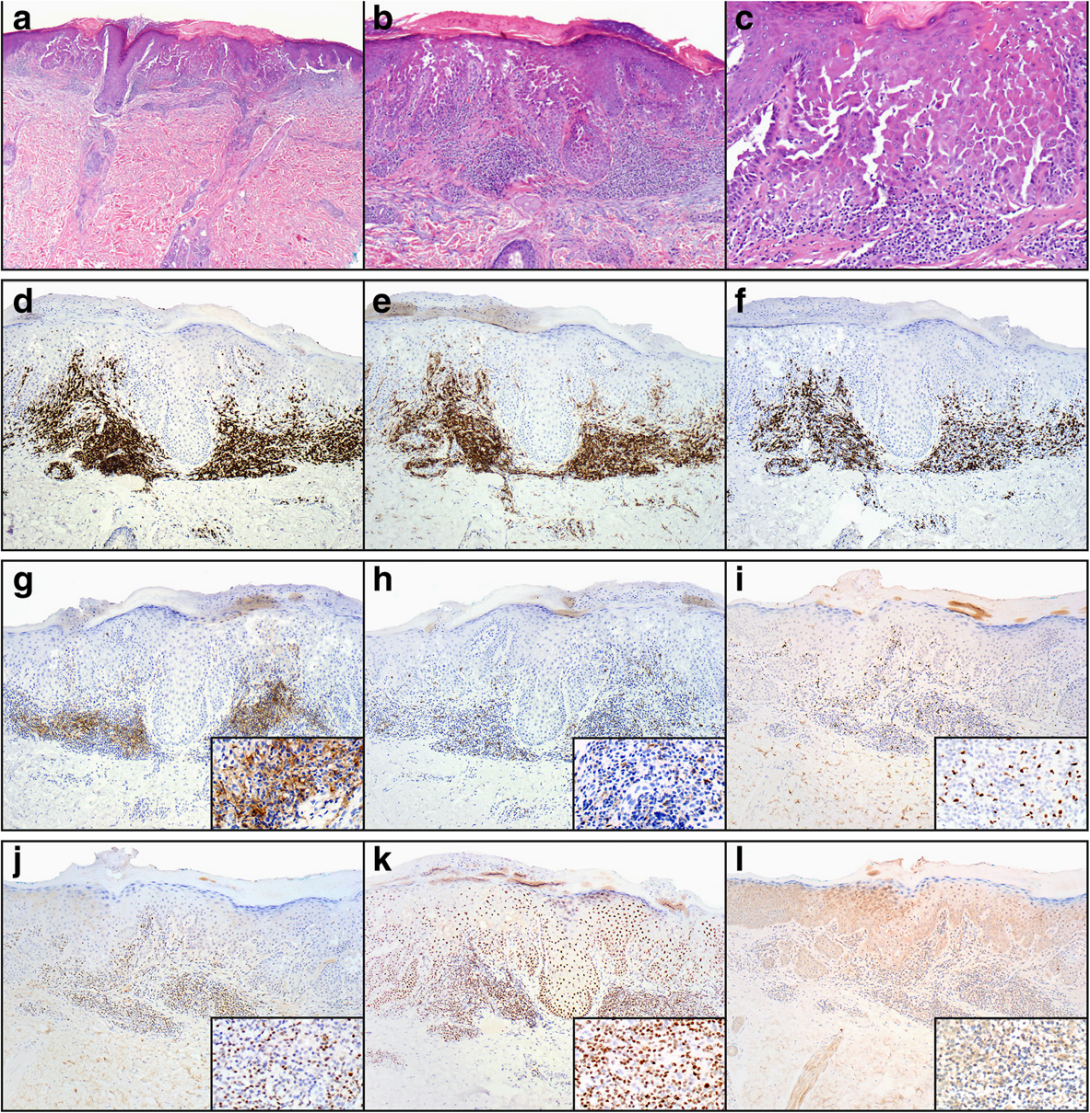
Histopathologic and immunophenotypic studies of ipilimumab-induced Grover’s disease. **a**-**c** Scanning magnification of skin with acantholytic dyskeratosis (**a**, H&E, 20×). Higher magnification reveals skin with suprabasal acantholysis and overlying dyskeratosis; (**b**, H&E, 100×) and (**c**, H&E, 40×). Immunohistochemical studies demonstrate a predominance of CD3+ T-cells (**d**, anti-CD3, 100×) comprised of a predominance of CD4+ T-cells (**e**, anti-CD4, 100×) over CD8+ T-cells (**f**, anti-CD8, 100×). Additional immunohistochemical studies demonstrate strong expression of PD-L1 by the inflammatory cells (**g**, anti-PD-L1, 100×; inset, 400×). Scattered cells express PD-1 (**h**, anti-PD-1, 100×; inset 400×). There is scattered nuclear expression of FoxP3 (**i**, anti-FoxP3, 100×; inset 400×) and T-beta (**j**, anti-T-Bet, 100×; inset, 400×) but strong diffuse nuclear expression of Gata-3 by the majority of the inflammatory cells as well as the overlying keratinocytes (**k**, anti-Gata-3, 100×; inset: 400×). Antibodies for RORgT are essentially negative in the infiltrate. There is strong background staining in the skin tissue (**l**, anti-RORgT, 100×; inset: 400×)

In July 2014, we evaluated the patient for progressive lung and liver metastases. He underwent a liver biopsy that confirmed metastatic melanoma with mutations in *BRAF* G466A and *RET W917*. Interestingly, he continued to have an extensive, erythematous, papular rash over his chest, arms, and back that was asymptomatic and had persisted since his previous ipilimumab therapy. He had no other oral ulcers or skin lesions. A skin biopsy was performed and histopathologic examination revealed suprabasal acantholysis with associated overlying dyskeratosis, consistent with Grover’s disease (Fig. 1a-c). The acantholysis extended to the spinous layer of the epidermis with some keratinocytes retaining partial attachment to one another. In addition, there was a superficial dermal mononuclear inflammatory infiltrate. Immunohistochemical (IHC) studies demonstrated a predominance of CD3+ T-cells with a predominance of CD4^+^ over CD8^+^ T-cells (Fig. 1d-f). Because anti-PD-1 therapy had not been FDA-approved at the time, he received systemic chemotherapy with disease progression. Considering his BRAF G466A mutation, we subsequently initiated single agent trametinib but his disease again progressed. Both chemotherapy and trametinib were initiated after the patient’s repeat skin biopsy.

**Fig. 2:**
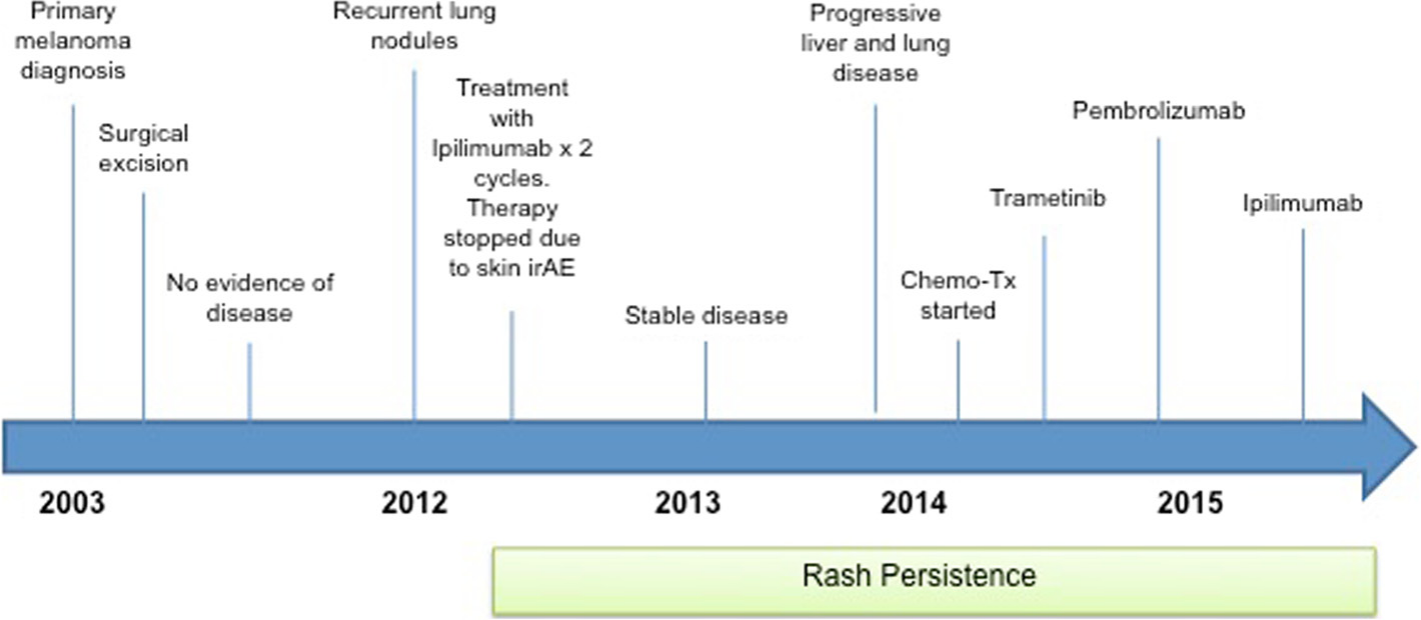
Chronologic timeline of patient’s clinical course

Thereafter, we started pembrolizumab at 2 mg/kg IV every three weeks. He tolerated therapy without significant toxicities. His previous rash symptoms remained stable during therapy with pembrolizumab. Unfortunately, after two cycles he continued to have rapid, progressive disease, and we discontinued therapy. Given the previous favorable response with ipilimumab, we re-initiated this therapy, but after one ipilimumab dose, he experienced grade 2 rash exacerbation with an intensely pruritic, papulovescular skin eruption on his upper back. A repeat biopsy confirmed Grover’s disease exacerbation. We started topical steroids and oral antihistamines with minimal symptom improvement. Thereafter, he received one additional ipilimumab dose but transitioned to hospice after demonstrating terminal, progressive disease. A treatment course timeline is included as Fig. 2.

## Discussion

Our case illustrates another example of ipilimumab-induced Grover’s disease (transient acantholytic dyskeratosis). In our patient, there was a direct relationship between onset of characteristic skin lesions with initial ipilimumab administration and lesion exacerbation after drug re-initiation. Remarkably, this immune finding does not seem to be a universal consequence of all CPI therapies as we observed stability of skin lesions with pembrolizumab, a PD-1 inhibitor. Moreover, ipilimumab-induced Grover’s disease appears to be a rare phenomenon given the paucity of reported cases. Nevertheless, it is important to emphasize that the histopathologic changes seen here are indistinguishable from that seen in sporadic Grover’s disease.

IrAEs are common in patients treated with ipilimumab. Phase 3 studies showed that the most common ipilimumab-associated irAEs were dermatologic, gastrointestinal, endocrine, and hepatic in nature [9, 10]. While the pathogenesis of irAEs is unclear, CD4^+^ T-cells have been shown to play a significant role [11]. Specifically, T helper 17 cells (Th17), which are a distinct lineage of CD4^+^ T-cells that produce characteristic cytokines like IL-17 and IL-22, have been implicated in a variety of autoimmune diseases and irAEs, particularly colitis [12]. Supporting this finding, pre-clinical studies have shown that blocking the CTLA-4:B7 interaction on antigen presenting cells and T cells, which is the mechanistic action of ipilimumab, potentiates Th17 cell differentiation in vivo and in vitro [12]. It is thought that blocking this CTLA-4:B7 interaction with ipilimumab, therefore, can potentiate Th17 mediated autoimmunity. In fact, a recent study reported on the correlation between IL-17 up-regulation, and the development of immune related colitis in patients treated with ipilimumab [13].

In our patient, IHC tissue analyses demonstrated marked CD3^+^ lymphocyte infiltration and a predominance of CD4^+^ over CD8^+^ T-cells (Fig. 1e-f). Additionally, there was moderate expression of PD-1/PD-L1 (Fig. 1g-h). Considering the above hypothesis, this finding was expected. However, analysis of protein expression using IHC of canonical transcription factors interestingly showed higher GATA3 over T-Bet and RORgT (Fig. 1j-l). (GATA3, or GATA-binding protein 3, is a Th2 transcription factor; T-bet, or T box transcription factor, is a Th1 transcription factor; and RORgT, or retinoic acid receptor-related orphan nuclear receptor, is a Th17 transcription factor). There was also minimal FoxP3 expression, indicating a limited contribution from Tregs (Fig. 1i). Taken together, these findings suggest that ipilimumab-induced Grover’s disease may result from action of Th2 cells with much less contribution from Th17. Moreover, the finding that pembrolizumab therapy did not exacerbate our patient’s Grover’s disease is consistent with pre-clinical data demonstrating that PD-1 inhibition induces Th1/Th17 responses while producing less Th2 type responses [14].

In light of our patient’s clinical history with supportive histologic and immunologic data, we believe that ipilimumab-induced Grover’s disease is an irAE that may be mediated by specific up-regulation and infiltration of Th2 rather than Th17 cells. This aligns with a previous report implicating IL-4, a known Th2 inducer, as a causative factor for Grover’s disease [7]. Having a deeper understanding of the characteristic immune response that mediates irAEs, and specifically dermatological irAEs in relation to Grover’s disease, may have a tremendous impact on how we manage and prevent them in a targeted way. As proof of concept, recent data has been published demonstrating the efficacy of IL-17A inhibition in the treatment of psoriatic arthritis [15]. Additionally, IL-6 receptor inhibitors are FDA approved for autoimmune disorders like rheumatoid arthritis and Crohn’s disease. This targeted approach to immune suppression through selective inhibition of auto-reactive cells like Th2/Th17 while minimally affecting the tumor specific Th1/CD8 cells, may have better efficacy in treating irAEs from CPI than generalized immune suppression with corticosteroids, which is the current practice. Furthermore, such an approach may preserve the cytotoxic effect of CPI. Obviously, this hypothesis can be confirmed only through prospective, randomized trials. Our report also illustrates the increasing complexity and wide variety of immune toxicities not experienced with traditional therapies.

## Conclusion

In conclusion, we report an addition case of Grover’s disease induced by ipilimumab. Interestingly, this dermatologic side effect did not appear with use of anti-PD-1 therapy in our patient. Although its exact pathogenesis is unknown, our immune analysis suggests a possible role of Th2 cells, which may warrant additional study.

## Abbreviations

CPI, checkpoint inhibitor; CT, computed tomography; CTLA-4, cytotoxic T-lymphocyte-associated protein 4; FDA, US Food and Drug Administration; IHC, immunohistochemistry; IL, interleukin; irAE, immune related adverse event; IV, intravenous; PD-1, programmed cell death protein 1
