# Erratum to: A case report of Grover’s disease from immunotherapy-a skin toxicity induced by inhibition of CTLA-4 but not PD-1

**DOI:** 10.1186/s40425-017-0208-7

**Published:** 2017-01-18

**Authors:** Marc Uemura, Faisal Fa’ak, Cara Haymaker, Natalie McQuail, Elizabeth Sirmans, Courtney W. Hudgens, Lydia Barbara, Chantale Bernatchez, Jonathan L. Curry, Patrick Hwu, Michael T. Tetzlaff, Adi Diab

**Affiliations:** 1 Department of Melanoma Medical Oncology, University of Texas-MD Anderson Cancer Center, Houston, TX USA; 2 Department of Pathology, University of Texas-MD Anderson Cancer Center, Houston, TX USA

## Erratum

Unfortunately, after publication of this article [1], it was noticed that the name of the second author was incorrectly displayed as Faa’k Faisal. The correct name is Faisal Fa’ak and can be seen in the corrected author list above. The original article has also been updated to correct this error.
